# Opportunities and challenges in recurrent diffuse podocytopathy post-transplantation: the critical value of the definition

**DOI:** 10.3389/fimmu.2026.1735978

**Published:** 2026-01-22

**Authors:** Rachel Nuccitelli, Amadea Toutoungis, Elena Martinelli, Simone Sanna-Cherchi, Astrid Weins, Heather K. Morris, Andrew S. Bomback, Ibrahim Batal

**Affiliations:** 1 Department of Surgery, Columbia University Irving Medical Center, New York, NY, United States; 2 Department of Pathology & Cell Biology, Columbia University Irving Medical Center, New York, NY, United States; 3 Department of Medicine, Division of Nephrology, Columbia University Irving Medical Center, New York, NY, United States; 4 Department of Pathology, Mass General Brigham and Harvard Medical School, Boston, MA, United States

**Keywords:** diffuse podocytopathy, kidney, pathology, recurrent disease, transplantation

## Abstract

Diffuse podocytopathy (DP) is a clinical and pathological entity, which comprises minimal change disease and primary focal segmental glomerulosclerosis (FSGS). It is characterized by diffuse podocyte foot process effacement resulting in nephrotic syndrome. Cumulative evidence supports that DP is a complex disease caused by circulating permeability factors. Following kidney transplantation, DP may recur and severely compromise graft survival. However, prior studies aiming to define immune and genetic factors implicated in disease recurrence have been limited by small cohorts and lack of utilizing stringent criteria to define DP. In this report, we briefly review the important advances made in understanding genomic and permeability factors involved in DP in the native kidney and in the transplant setting, focusing on anti-nephrin antibodies. We stress the importance of applying stringent criteria to define patients at risk of post-transplant recurrence and share our experience in a cohort of 281 consecutive kidney transplant recipients with native kidney failure attributed to FSGS or other forms of DP. Applying strict clinicopathologic criteria combining nephrotic syndrome and diffuse foot process effacement at the time of native kidney biopsy to define DP markedly increased recurrence rate from 9% to 36%. Excluding selected patients with monogenic forms of FSGS and those with high-risk *APOL1* genotypes further increased recurrence rate to 54%. In conclusion, an accurate diagnosis of DP in the native kidney is crucial to further our understanding of genomic and immunologic predictors of DP recurrence and to ultimately support the development of prophylactic or therapeutic regimens to improve allograft outcomes.

## Introduction

Podocytes are highly specialized, terminally differentiated epithelial cells with limited regenerative capacity (1). Because they are the main regulators of the glomerular filtration barrier, podocyte injury can lead to proteinuria. Numerous insults can cause podocyte injuries (podocytopathies). These include, but are not limited to, focal segmental glomerulosclerosis (FSGS), minimal change disease (MCD), immune complex-mediated glomerulopathies, dysproteinemia, and a variety of systemic diseases (2).

Although FSGS is regarded as the “prototype” for podocytopathies, it is not a distinct disease entity. Instead, FSGS represents a late histologic manifestation of different injuries resulting from diverse insults that cause significant podocyte depletion, typically exceeding 20% (3, 4). Given the reduced regenerative capacity of podocytes, this loss leads to segmental obliteration of glomerular capillaries, hyaline accumulation, and adhesion to Bowman’s capsule, collectively referred to as segmental sclerosis. Eventually, segmental sclerosis further progress to global glomerulosclerosis.

Given the ambiguity of the term “FSGS”, which has become deeply embedded in medical literature, the Kidney Disease Improving Global Outcomes (KDIGO) proposed classifying FSGS into four distinct categories: 1) Primary (idiopathic), typically associated with nephrotic syndrome and diffuse foot process effacement (FPE); 2) Secondary, the most common category, often adaptive in nature, but can also include other secondary causes, such as viral infections and medications. Classically, secondary FSGS lacks nephrotic syndrome and diffuse FPE; 3) Genetic, linked to a growing list of monogenic mutations (such as *NPHS1, NPHS2, WT1*, *TRPC6, INF2, PAX2, COL4A*, *etc.*), which initially thought to lack the combination of nephrotic syndrome and diffuse FPE; and 4) Undetermined, where no clear etiology is identified (5).

While accurate classification of FSGS requires an integrative approach that combines clinical history, laboratory findings, histopathologic evaluation, and often genetic testing (2), KDIGO classification remains less than ideal. The distinction between primary FSGS and undetermined FSGS is often vague. Moreover, many cases are still “unresolved” under such classification. For example, *APOL1* high-risk genotypes, which are commonly encountered in subjects with recent African ancestry, are not currently categorized under the genetic forms of FSGS.

## Concept of diffuse podocytopathy

**Figure 1:**
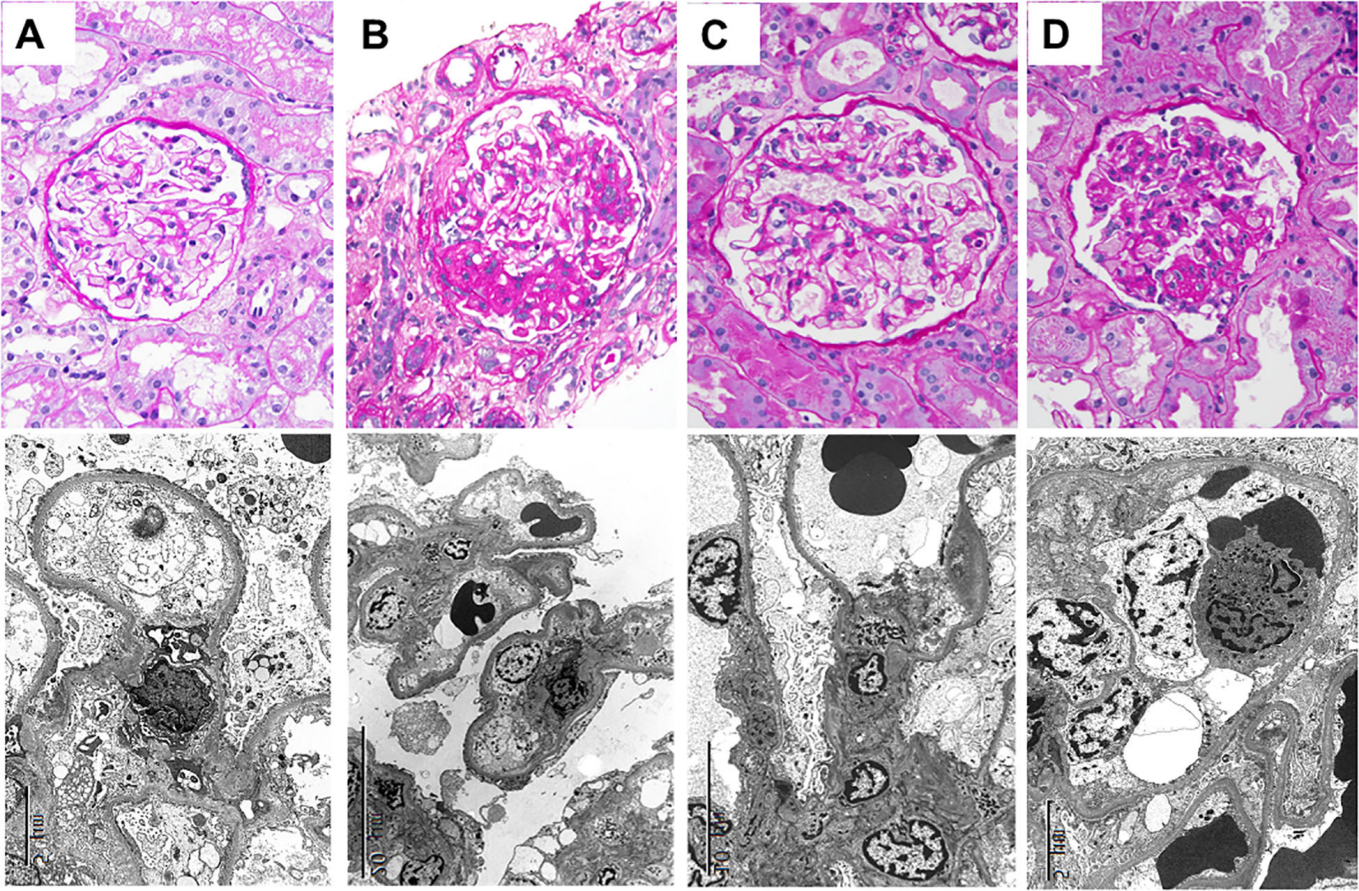
Kidney biopsy findings from a patient who developed minimal change disease followed by focal segmental glomerulosclerosis in both native kidney as well as in the kidney allograft in the form of recurrent disease **(A)** Patient presented with nephrotic syndrome (urinalysis showing 4+ albumin and serum albumin of 2.1 g/dL). A native kidney biopsy showing a glomerulus with no significant abnormalities by light microscopy (upper panel, Periodic acid–Schiff, original magnification, ×400.). Ultrastructural examination reveals foot process effacement involving 90% of the glomerular capillary surface areas (lower panel, electron microscopy, original magnification, ×6,000) **(B)** About 20 months later, the patients presented again with nephrotic syndrome after failing to respond to prednisone (urine protein-to-creatinine > 15 g/g and serum albumin of 1.8 g/dL). A follow-up native kidney biopsy showing a glomerulus with segmental sclerotic lesion (upper panel, Periodic acid–Schiff, original magnification, ×400.). Ultrastructural examination reveals foot process effacement involving 70% of the glomerular capillary surface areas (lower panel, electron microscopy, original magnification, ×3,000) **(C)** Three days post-transplantation, the patient presented with urine protein-to-creatinine of 7.8 g/g and serum albumin of 2.2 g/d. L An allograft biopsy showing a glomerulus with no significant abnormalities by light microscopy (upper panel, Periodic acid–Schiff, original magnification, ×400.). Ultrastructural examination reveals foot process effacement involving 90% of the glomerular capillary surface areas (lower panel, electron microscopy, original magnification, ×5,000). **(D)** About 19 weeks later, the patients presented with persistent nephrotic range proteinuria despite treatment with plasmapheresis (urine protein-to-creatinine of 5.6 g/g). A follow-up allograft kidney biopsy showing a glomerulus with segmental sclerotic lesion (upper panel, Periodic acid–Schiff, original magnification, ×400.). Ultrastructural examination reveals foot process effacement involving 75% of the glomerular capillary surface areas (lower panel, electron microscopy, original magnification, ×6,000).

MCD is believed to be caused by circulating factors that induce diffuse and global injury to the glomerular filters, leading to temporary loss of compensation, and the development of nephrotic syndrome. This concept also applies to primary FSGS, which is additionally characterized by the development of segmental sclerosis. It is plausible that once a threshold of podocyte depletion is crossed due to prolonged injury, severe damage, and/or a genetically vulnerable background, segmental sclerosis may develop later (6, 7). Indeed, many cases have documented progression from MCD to FSGS over time (8–11) (**Figures 1A, B**). This transition is naturally observed in the transplant setting, where recurrent primary FSGS initially presents with MCD-like features and evolves into segmental sclerosis within weeks (**Figures 1C, D**). It is therefore logical to group MCD and primary FSGS, under the term “diffuse podocytopathy” (DP) (12).

## Recent advantages in understanding DP in the native kidney

Cumulative evidence supports that DP in the native kidney is associated with circulating permeability factors. Recent discoveries have enhanced our understanding of this concept. Anti-nephrin autoantibodies have been shown to play an important role in MCD (13–15) and primary FSGS (15, 16) (**Supplementary Figure S1**). Beyond their diagnostic utilities, testing for anti-nephrin antibodies may be important in monitoring disease activity and informing therapeutic strategies (14, 15, 17, 18).

Additional permeability factor candidates include those targeting other slit diaphragm components, such as Podocin and Kirrel1 (19), other antibodies, such as anti-CD40 antibodies (20), and non-antibody circulating permeability factors, such as cardiotrophin-like cytokine-1 (CLC-1) and soluble urokinase type plasminogen activator receptor (suPAR) (21).

Genome wide association studies (GWAS) have uncovered additional layers related to the pathogenesis of DP. GWAS studies in children with steroid-sensitive nephrotic syndrome (SSNS) have identified several HLA and non-HLA susceptibility loci (22, 23). Recently, researchers successfully constructed a polygenic risk score that could explain earlier onset of such nephrotic syndrome (24).

## Challenges in studying DP in the native kidney

Despite these recent discoveries, studying DP remains challenging. Limitations for the assessment of anti-nephrin antibodies include the lack of commercially available serological testing and the variable enzyme-linked immunosorbent assays (ELISA) currently in use for research purposes. Some ELISA use recombinant peptides produced in human embryonic kidney cells while others use peptides made in a murine cell line (13, 25). The latter may diminish ELISA specificity due to serum reactivity with dietary circulating antibodies directed against certain non-human glycan epitopes (including, but not limited to, alpha-gal) on murine nephrin, preventing meaningful comparison between different studies. Experience in other permeability factors is still very preliminary. Regarding GWAS studies, published reports are often enriched with children diagnosed with SSNS, who are frequently identified clinically without a kidney biopsy.

Yet, a leading problem in studying DP might be related to potential contamination of the cohorts by secondary and genetic forms of FSGS. To increase the purity of the studies, De Vriese et al. from Mayo Clinic proposed using a standard definition that is derived from the pathophysiology of this disease: presence of nephrotic range proteinuria (≥3.5 g/g), low serum albumin (≤3.5 g/dL) and near-complete FPE (≥80%) (26). Each of these criteria by itself lacks specificity. For example, nephrotic range proteinuria can be encountered in cases with considerable glomerular injuries and/or substantial glomerulosclerosis. Similarly, hypoalbuminemia can be observed in many conditions, including hepatic dysfunction. Even the extent of FPE may be dependent on the scoped glomeruli, where injured glomeruli (such as segmentally sclerotic or hypo-perfused) may show extensive FPE (**Supplementary Figure S2**). Glomeruli present at the edge of the sections may also show denudation of the basement membrane and artificial loss of foot processes. However, when applied together, these three criteria appear to provide reasonable specificity for diagnosing DP (27). Unfortunately, these criteria were often not applied.

## Challenges in studying post-transplant recurrence of DP

Whereas studying DP in the native kidney is difficult, studying recurrent DP in the kidney allograft is even more challenging. The reasons include considerably smaller samples, and high incidence of adaptive FSGS in the allograft (typically one kidney is transplanted and this kidney is continuously exposed to various types of injuries). More remarkably, incorrect labeling of the native kidney disease as DP is readily transferred to the kidney allograft and using the general term “FSGS” without further classification of the lesion, would often be carried over to the allograft and treated wrongly as “primary FSGS/DP”.

These obstacles have led to a substantial variability in the data. While the largest study to date reported a recurrence rate of 32% in primary FSGS (28), different studies have reported widely variable prevalence rates of recurrent FSGS, ranging from 15% (29) to 64% (30). Published studies often lacked a precise definition of primary FSGS/DP in the native kidney and the findings raised concerns over potential contamination of the studied cohorts by non-primary FSGS. For example, several published reports have shown that recipients of White race may have a higher risk of disease recurrence (28, 31, 32), which may instead reflect the increased prevalence of *APOL1* high-risk genotypes in non-White recipients (11). While *APOL1* high-risk genotypes can drive nephrotic syndrome in the native kidney (33), they rarely recur as DP after transplantation. A large study found that a high body mass index (BMI) was protective against recurrent disease (28). This finding may also suggest potential contamination of the cohort by adaptive FSGS secondary to obesity, which does not recur after transplantation as DP.

A thorough evaluation of the clinical-pathological courses in the native disease may enhance correct labeling of these cases and, thus, may give insight on risk of post-transplant recurrence. For example, one study has shown that initial response to steroids followed by steroid resistance in the native kidney was a predictor for disease recurrence post-transplantation (34). Another study has demonstrated that the presence of minimal change disease on initial native kidney biopsy was associated with increased risk of post-transplant recurrence (35).

## Permeability factors and recurrent DP

A Japanese multicenter study included 14 pediatric patients with presumed primary FSGS (11 recurrent and 3 non-recurrent) and 8 with genetic forms of FSGS (25). Patients with recurrent disease showed punctate IgG that colocalized with nephrin. Positive circulating anti-nephrin antibodies were detected in all patients with recurrent FSGS, one third of patients without recurrence, and none of the patients with genetic forms of FSGS. Some limitations of this study included restriction of the cohort to pediatric Asian population, small sample size, ELISA methodology using recombinant murine nephrin that may have led to some non-specific bindings, and the lack of a standardized timing of serum sampling, with assessment for anti-nephrin antibodies before transplantation in some patients and after transplantation in others. For addressing the importance of anti-nephrin antibodies in stratifying patients for risk of disease recurrence, the assessment of pre-transplant anti-nephrin antibodies is prudent.

We performed a retrospective multicenter study, which included 38 kidney transplant recipients with native kidney failure attributed to DP with available pre-transplant sera (21 with recurrence and 17 without recurrence) (36). DP was defined according to the criteria proposed by De Vriese et al. (26), and a validated indirect ELISA (using recombinant human nephrin) with predetermined threshold to define positive and negative cases was used (13). This study found that positive pre-transplant anti-nephrin antibodies had 100% specificity and 38% sensitivity in predicting recurrent DP. As expected, allograft biopsies from patients with anti-nephrin antibodies showed punctate IgG colocalizing with nephrin. Most biopsies with recurrent DP in patients who were negative for anti-nephrin antibodies revealed punctate nephrin staining reflecting nephrin redistributed secondary to podocyte injury, which was associated with different staining patterns for IgG, including punctate IgG without significant colocalization with nephrin, or negative IgG (36). The latter findings raise the possibility of the presence of other non-nephrin antibodies as well as non-antibody permeability factors Amongst others, limitations of this study included enrichment of the cohort with White recipients, relatively small sample size, and the lack of genetic testing in these patients.

## Genetic factors and recurrent DP

GWAS studies in recurrent DP are generally lacking. Discovery studies are extremely difficult to conduct due to small sample size. Translating genetic insights from studies in the native kidney can be tricky, since those cohorts are mostly composed of children with SSNS. In contrast, adult patients and those with steroid resistant nephrotic syndrome, which are undersampled in published GWAS studies, are more likely to progress to native kidney failure and undergo kidney transplantation.

As a result, our understanding of hereditary risk factors for DP recurrence is extremely limited. Some studies have suggested associations between recurrent DP and potential permissive HLA antigens in both recipients and donors (11, 37). However, these studies were limited by lack of high-resolution HLA typing, and small sample sizes or relying on registry data. Future GWAS studies are necessary to assess genetic factors involved in recurrent DP, while properly controlling for genetic ancestry.

## Precise definition of DP in the native kidney is crucial to stratify kidney transplant recipients according to the risk of recurrence

While the recurrence rate of DP is highly variable (29, 30), important confounders that may affect recurrence rate include contamination of the cohorts by non-primary FSGS, which do not recur as nephrotic syndrome. Whether applying the stringent criteria by De Vriese et al. (26) to define DP in the native kidney improves stratification of patients at risk of recurrence after transplantation remains unknown.

To address this question, we retrospectively analyzed patients who underwent kidney transplantation at CUIMC between 2005 and 2024 with native kidney failure attributed to FSGS or other forms of DP, under approval by the Institutional Review Board of Columbia University. The cohort included 281 patients: 270 with native kidney biopsy labeled as FSGS (2 labeled as C1q nephropathy), 9 with MCD (including 6 who later developed FSGS on follow-up biopsy), and 2 with nephrotic syndrome not otherwise specified. When all cases were included, the recurrence rate of DP (positive predictive value) was 9% (**Table 1**). Because no diagnostic criteria were applied, specificity could not be evaluated. Restricting the analysis to patients with nephrotic range proteinuria (≥3.5 g/g) increased recurrence rate to 17% with a specificity of 38%, and further restricting selection to patients meeting full nephrotic syndrome (proteinuria ≥3.5 g/g and serum albumin ≤3.5 g/dL) raised recurrent rate to 29% with specificity of 66%. Applying all clinical and pathological criteria of De Vriese et al. (proteinuria ≥ 3.5 g/g, serum albumin ≤ 3.5 and FPE ≥80%) increased the recurrence rate to 36% with a specificity of 82%. Despite improved specificity and positive predictive value, high negative predictive values (NPV) (98–99%) were observed using different defining features (**Table 1**).

**Table 1:** Sensitivity, specificity, positive predictive value, and negative predictive value of diffuse podocytopathy definition in predicting recurrent disease.

Diagnostic group	Recurrent DP (n)	No recurrence (n)	Total (n)
All DP	24	257	281
NRP	18	86	104
No NRP	1	52	53
NS	17	42	59
No NS	1	81	82
NS + ≥80% FPE	15	27	42
No NS or <80% FPE	2	119	121
NS + ≥80% FPE excluding resolved DP from genetic causes ^*^	15	13	28
No NS or <80% FPE	2	119	121

NRP, nephrotic range proteinuria (≥3.5 g/g); NS, nephrotic syndrome (proteinuria (≥3.5 g/g) and serum albumin ≤3.5 g/dl); FPE, foot process effacement, PPV, positive predictive value; NPV, negative predictive value; Total number of cases included depends on the available data in the native kidney that can be used to classify the patients.

When only urine dip stick was available; 4+ was considered “nephrotic range” while 0-2+ were considered “sub-nephrotic”; values with 3+ were excluded.

^*^Genetic exclusions (e.g., high-risk *APOL1* (n=9), *NPHS2* (n=3), *COL4A5* (n=2). Mutations shown in **Supplementary Table S1**.

• All DP: Sensitivity = 100%; Specificity = 0%; PPV = 9%; NPV not applicable.

• NRP vs. No NRP: Sensitivity = 95%; Specificity = 38%; PPV = 17%; NPV = 98%.

• NS vs. No NS: Sensitivity = 94%; Specificity = 66%; PPV = 29%; NPV = 99%.

• NS + ≥80% FPE vs. others not meeting definition: Sensitivity = 88%; Specificity = 82%; PPV = 36%; NPV = 98%.

NS + ≥80% FPE excluding positive genetic tests vs. others: Sensitivity = 88%; Specificity = 90%; PPV = 54%; NPV = 98%.

When only urine dip stick was available and both 3+ and 4+ were considered “nephrotic range” while 0-2+ were considered “sub-nephrotic”, the specificity and PPV values were minimally decreased using some of the above definitions: All DP [Sensitivity = 100%; Specificity = 0%; PPV = 9%; NPV not applicable]; NRP vs. No NRP [Sensitivity = 95%; Specificity = 36%; PPV = 16%; NPV = 98%]; NS vs. No NS [Sensitivity = 94%; Specificity = 63%; PPV = 27%; NPV = 99%]; NS + ≥80% FPE vs. others not meeting definition: Sensitivity = 88%; Specificity = 81%; PPV = 35%; NPV = 98%]; NS + ≥80% FPE excluding positive genetic tests vs. others [Sensitivity = 88%; Specificity = 89%; PPV = 52%; NPV = 98%].

Whereas monogenic forms of FSGS have initially been assumed to have subnephrotic proteinuria and focal foot process effacement, recent reports have shown that they can present with nephrotic syndrome and diffuse foot process effacement (35, 38, 39). Similarly, a proportion of patients with FSGS attributed to *APOL1* high-risk genotypes may have nephrotic syndrome (40). Nevertheless, genetic forms of FSGS, irrespective of the presence or absence of nephrotic syndrome or diffuse FPE, do not typically recur after transplantation (41, 42). Therefore, we next assessed whether genetic studies might further improve recurrence risk stratification. Of the 42 patients who met De Vriese et al. criteria for DP, genetic testing was performed in 30 (**Supplementary Material**), and a genetic cause was identified in 14, including high-risk *APOL1* genotypes (n=9), *NPHS2* mutations (n=3), and *COL4A5* mutations (n=2) (**Supplementary Table S1**, **Supplementary Figures S3A, B**). After excluding these resolved cases, recurrence rate increased to more than half (54%) and specificity increased to 90% (**Table 1**). Limitations of this attempt include its retrospective nature and lack of available laboratory and/or electron microscopy data at native kidney biopsies in some patients. The genetic testing approaches and modalities were also variable. Some patients did not undergo genetic testing at all, a subset of patients had genetic testing using various commercial panels selected by treating clinicians, and some were tested for research purposes. The latter assays also varied from *APOL1* genotyping only, to broad assays such as exome and genome sequencing (**Supplementary Material**). Despite these limitations, our findings support that integrating genetic testing with stringent clinical and pathologic criteria to define DP in the native kidney is informative in identifying patients at high risk of DP recurrence.

## Future opportunities

Understanding the immunogenetic foundations of DP in the native kidney is important for anticipating, preventing, and treating disease recurrence in the transplant setting. While recurrent DP is associated with detrimental effects on allograft survival, our understanding of risk factors for disease recurrence remains limited. GWAS studies focusing on adult patients with SRNS are crucial, as these individuals are more likely to develop native kidney failure and undergo transplantation. In the transplant settings, accurate documentation of laboratory and kidney biopsy results at the time of diagnosing DP or FSGS in the native kidney is critical. Applying stringent criteria is a necessary first step to filter-out adaptive forms of FSGS, which do not recur as DP after transplantation. Conducting genetic studies on patients who pass the clinical and pathological filtration process will further refine patient selection by excluding those with monogenic or *APOL1-*associated forms of the disease, both of which typically do not recur post-transplantation as DP.

Future studies would benefit from assessing pre- and post-transplantation sera for anti-nephrin antibodies and other potential permeability factors. Confirmatory findings in the tissue can offer additional validation. Molecular studies would benefit from evaluating post-reperfusion and follow-up kidney transplant biopsies to capture early and serial changes that occur after exposure of donor kidney to circulating permeability factors. Clinical studies that address the effects of B cell and plasma cell depletion would be important in improving our approach in preventing and treating recurrent DP. In summary, multi-center studies that benefit from donor and recipient genomic profiling, along with serial serologic and multi-omics studies, will improve our understanding of the pathogenesis of recurrent DP.

## Conclusion

In conclusion, current evidence suggests that recurrent DP can be mediated by anti-nephrin antibodies and as well as additional antibody and non-antibody permeability factors. Comprehensive genetic studies, especially GWAS will be valuable in deepening our understanding of the pathogenesis of recurrent DP, especially in patients who are negative for anti-nephrin antibodies. Proper documentation of clinical and biopsy parameters in the native kidney and a precise definition of DP in the native kidney as well as in the allograft is critical for prognostication, management, and advancing our understanding of recurrent DP after kidney transplantation.

## Data Availability

Clinical data can be provided from the corresponding author with reasonable request after approval from Institutional Review Board. The CUIMC dataset includes genome or exome sequencing from individuals enrolled at CUIMC and at external national and international institutions, as well as consortia. Exome sequencing data from the Columbia University Genetic Studies of Chronic Kidney Disease (CKD) (Accession: phs001828.v1.p1, https://nam02.safelinks.protection.outlook.com/?url=https%3A%2F%2Fwww.ncbi.nlm.nih.gov%2Fprojects%2Fgap%2Fcgi-bin%2Fstudy.cgi%3Fstudy_id%3Dphs001828.v1.p1&data=05%7C02%7Cib2349%40cumc.columbia.edu%7C458844a7340b4fe0932c08de3cbec577%7Cb0002a9b0017404d97dc3d3bab09be81%7C0%7C0%7C639014987228908661%7CUnknown%7CTWFpbGZsb3d8eyJFbXB0eU1hcGkiOnRydWUsIlYiOiIwLjAuMDAwMCIsIlAiOiJXaW4zMiIsIkFOIjoiTWFpbCIsIldUIjoyfQ%3D%3D%7C0%7C%7C%7C&sdata=f3X7JExCBAZbyaC%2FB38KTECZ9Py4Gcf1TWgXUBembuE%3D&reserved=0), is deposited in dbGaP. The data of the few patients described in this manuscript are available in the article and in the **Supplementary Information** and upon request from SSC and the corresponding author. The analyses described here do not reflect exome- or genome-wide analysis of the data but only a targeted analysis of the APOL1 variants and Mendelian FSGS genes. As such, a complete genome-wide analysis of these data has not yet been conducted, and the full data will be deposited in dbGaP at completion of the genome-wide analyses.
