# Supplementary material for: Opportunities and challenges in recurrent diffuse podocytopathy post-transplantation: the critical value of the definition
Source: Front Immunol. 2026 Jan 22;17:1735978. doi: 10.3389/fimmu.2026.1735978 (PMC12872477; doi:10.3389/fimmu.2026.1735978)
Supplement: Supplementary file 1 [file Supplementaryfile1.pdf]

Supplementary material

Genetic testing

Genetic testing was performed in 30 cases meeting De Vriese et al. criteria for diffuse podocytopathy (DP; nephrotic syndrome and foot process effacement involving ≥80% of the glomerular capillary surface area). Genetic testing was performed by different methods varying from part of clinical care (n=7), *APOL1* genotyping from residual tissue (n=1), genotyping by high-density SNPs (n=11), and whole exome/genome sequencing (n=11).

Genetic testing as part of clinical work-up

Genetic testing was performed as part of clinical care for 7 patients where data were interpreted in medical records as a potentially disease-causing variant. Specifically, 5 cases underwent targeted genetic testing using the Renasight panel (Natera, San Carlos, CA), a next-generation sequencing–based assay that screens for pathogenic variants in >380 genes associated with chronic kidney disease (CKD), including genes implicated in focal segmental glomerulosclerosis (FSGS), incorporating *APOL1*. Natera testing was conducted in a CLIA-certified laboratory following standard clinical sequencing protocols, with variants classified according to American College of Medical Genetics (ACMG) guidelines (1). Positive sequencing results were returned as reports. One case underwent targeted genetic testing using the 57-gene panel for nephrotic syndrome and FSGS from Invitae (San Francisco, CA). One case underwent targeted genetic testing using the 4-gene (*INF2, ACTN4, TRPC6, NPHS2*) panel from Athena Health (Boston, MA).

APOL1 genotyping from residual kidney biopsy

One case underwent genotyping for *APOL1* from formalin-fixed paraffin-embedded residual tissue at Arkana laboratory as previously described (2). Briefly, Taqman PCR was performed on a QuantStudio 5 Real-Time PCR system, genotyping data were evaluated using QuantStudio Design and Analysis software, and allelic discrimination plots were used to assess the call results.

Sample Collection and Informed Consent for Research

In 22 cases, genomic DNA was isolated using standard protocols from peripheral whole blood leukocytes drawn by venipuncture following informed consent. DNA extraction was performed using the QIAamp DNA Mini Kit (Qiagen) according to the manufacturer's protocol. DNA concentration and purity were assessed using the Qubit fluorometer.

Genotyping Data

Genotyping was performed on 11 cases using multiple versions of the Illumina Multi-Ethnic Global Array (MEGA) chips, including MEGA 1.0, MEGA 1.1, and MEGAEX. From these datasets, APOL1 risk variants G1 (rs73885319) and G2 (rs71785313) were extracted to define high-risk and low-risk genotypes.

Exome/Genome Data

A total of 7 cases underwent exome sequencing using one of the following capture kits: the IDTERPv1 exome capture kit (n=1), the Roche V1 or V2 exome capture kits (n=5), and the Agilent V4 exome capture kit (n=1). An additional 4 cases were subjected to whole-genome sequencing (WGS). FASTQ and BAM files were processed, curated, and analyzed at the Institute for Genomic Medicine (IGM) at CUIMC.

Variant Annotation and Interpretation from Exome and Genome Sequencing

The exome and genome sequences were analyzed using an in-house designed pipeline to identify disease-associated variants in accordance with the ACMG guidelines for clinical variant interpretation (1). Variant filtering was performed to require a quality score > 50, quality by depth score ≥2, genotyping quality score ≥20, mapping quality score ≥40, coverage ≥10, alternate read percentages was within the range of 0.3 and 0.7 for heterozygous genotypes. For variant prioritization, we used Varsome (https://varsome.com/) web-based platform that implement the ACMG guidelines as a first-pass screen to predict an ACMG verdict for each uploaded variant for further clinical variant interpretation and genotype-phenotype correlation for all the genes queried (3, 4). We used the following criteria to define a positive genetic finding for our clinical research variant adjudication. First-tier positive findings were considered if the genotype was already reported as pathogenic or likely pathogenic in ClinVar (5) or classified as pathogenic or likely pathogenic by strict ACMG criteria via individual variant curation in Varsome. Since missense variants and variants never observed in public databases such as gnomAD rarely meet ACMG P/LP criteria and are often classified as US (unknown significance), in order to define our second-tier positive genetic finding, we used the following criteria: absent of exceedingly rare in public databases as well as in our in-house 11,818 multiethnic population controls from the IGM and excluding variants known or predicted to have low likelihood of pathogenicity (benign or likely benign in the Polyphen and ClinVar databases, and/or with REVEL score <0.3, PrimateAI score <0.6) (6, 7). *APOL1* risk alleles were determined by extracting rs73885319 (G1) and rs71785313 (G2) genotypes from Exome/Genome Data using the ATAV platform (8).

Supplementary Table S1: Genetic variants pathogenic for FSGS

Subjects	Method used	Genetic variant
Subject #1	Invitae 57-gene panel for nephrotic syndrome and FSGS	*NPHS2*: homozygous variants c.538G>A (p.Val180Met)
Subject #2	Athena Health 4-gene panel	*NPHS2:* heterozygous variants c.851C>T (p.Ala284Val) and c.686G>A (p.Arg229Gln)
Subject #3	*APOL1* genotyping from residual tissue	*APOL1* high-risk genotype (G1/G2)
Subject #4	Genotyping by high density SNPs	*APOL1* high-risk genotype (G2/G2)
Subject #5	Genotyping by high density SNPs	*APOL1* high-risk genotype (G1/G2)
Subject #6	Genotyping by high density SNPs	*APOL1* high-risk genotype (G1/G2)
Subject #7	Genotyping by high density SNPs	*APOL1* high-risk genotype (G1/G1)
Subject #8	Genotyping by high density SNPs	*APOL1* high-risk genotype (G1/G1)
Subject #9	Genotyping by high density SNPs	*APOL1* high-risk genotype (G1/G1)
Subject #12	Genotyping by high density SNPs	*APOL1* high-risk genotype (G1/G1)
Subject #11	Exome sequencing	*COL4A4*: heterozygous variant c.213_239del (p.Pro72_Gly80del) and heterozygous variant c.4288G>A (p.Gly1430Arg)
Subject #12	Exome sequencing	*APOL1* high-risk genotype (G1/G2)
Subject #13	Genome sequencing	*COL4A4:* heterozygous variant (c.1022G>A, pGly341Asp)
Subject #14	Exome sequencing	*NPHS2:* c.928G>A (p.Glu310Lys) and c.686G>A (p.Arg229Gln) het

Supplementary Figure S1:

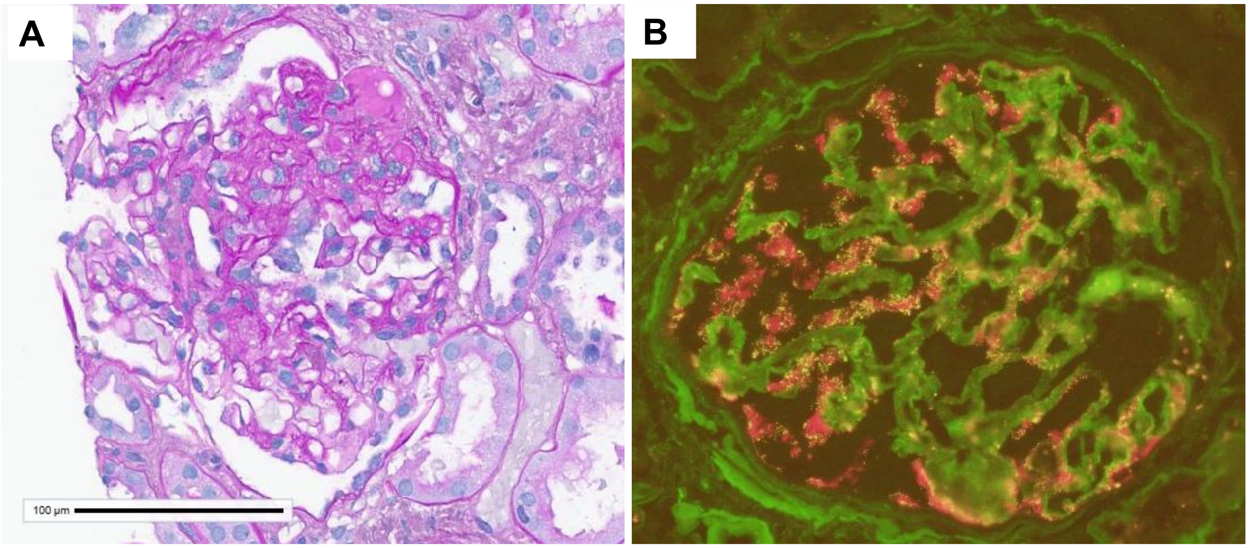

Patient presented with nephrotic syndrome (urine protein-to-creatinine of 18.8 g/g and serum albumin of 2.2 g/dL) (**A)** Native kidney biopsy showing a glomerulus with segmental sclerotic lesion (Periodic acid–Schiff, original magnification, ×600.) **(B)** immunofluorescence shows punctate IgG (green) and granular nephrin (red), which show marked overlapping (yellow).

Figure S2

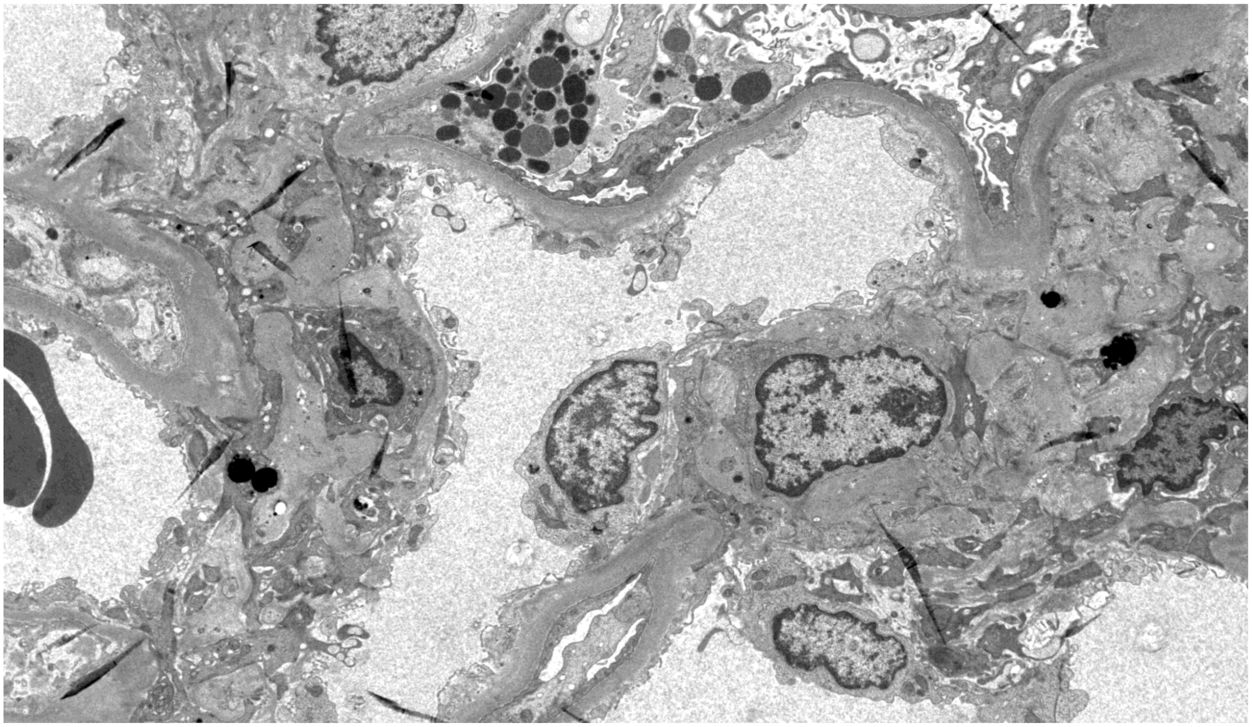

A patient presented with renal insufficiency, subnephrotic proteinuria (1.2 g/day) and normal serum albumin of 4.2 g/dL. The glomerulus submitted for electron microscopy is mildly ischemically retracted and shows extensive foot process effacement (electron microscopy, original magnification, ×2,500)

Figure S3:

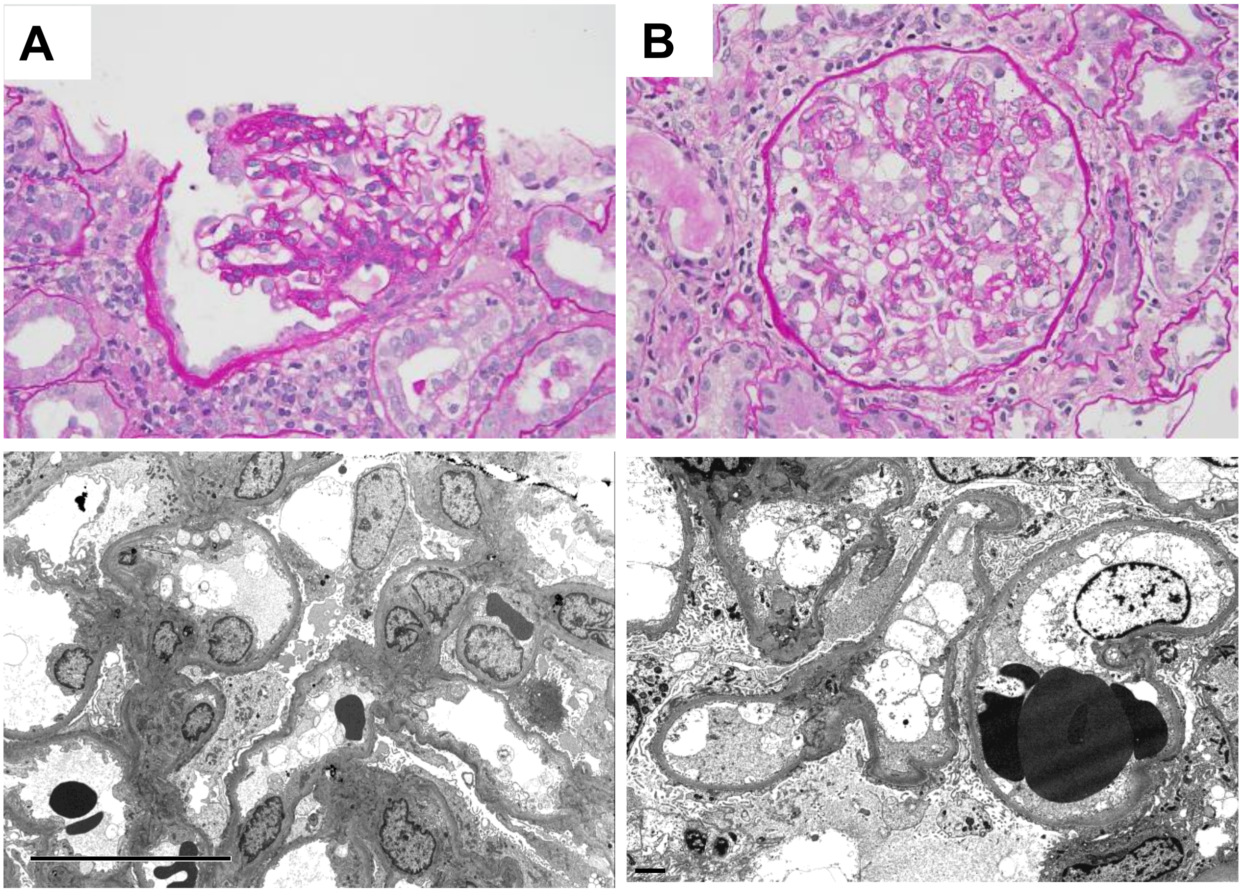

(A) A 21 year-old patient presented with nephrotic syndrome (proteinuria: 9 g/day and serum albumin of 2.1 g/dL). A native kidney biopsy showing a glomerulus with segmental sclerosis (upper panel, Periodic acid–Schiff, original magnification, ×400.). Ultrastructural examination reveals foot process effacement affecting 90% of the glomerular capillary surface areas (lower panel, electron microscopy, original magnification, ×3,000). Genetic testing by high density SNPs reveals *APOL1* high-risk genotypes (G2/G2). This patient did not develop recurrence post-transplantation **(B)** A 23 year-old patient presented with nephrotic syndrome (proteinuria: 16 g/day and serum albumin of 1.5 g/dL). A native kidney biopsy showing a glomerulus with segmental sclerosis (upper panel, Periodic acid–Schiff, original magnification, ×400.). Ultrastructural examination reveals foot process effacement affecting 90% of the glomerular capillary surface areas (lower panel, electron microscopy, original magnification, ×4,000). Exome sequencing revealed compound heterozygous mutations in the *NPHS2* gene [c.928G>A (p.Glu310Lys) and c.686G>A (p.Arg229Gln)]. This patient did not develop disease recurrence post-transplantation

Acknowledgment:

Arkana laboratories had no role in the study design, the interpretation of data, or the decision to publish results.

References

1. Richards S, Aziz N, Bale S, Bick D, Das S, Gastier-Foster J, et al. Standards and guidelines for the interpretation of sequence variants: a joint consensus recommendation of the American College of Medical Genetics and Genomics and the Association for Molecular Pathology. Genet Med. 2015;17(5):405–24.
2. Batal I, Mikhailov AV, Husain SA, Nuccitelli RA, Zanoni F, Liu L, et al. Association of Collapsing Glomerulopathy with Donor Apolipoprotein L1 Risk Variants in Kidney Allografts from Black Donors. Clin J Am Soc Nephrol. 2025;20(9):1268–76.
3. Groopman EE, Marasa M, Cameron-Christie S, Petrovski S, Aggarwal VS, Milo-Rasouly H, et al. Diagnostic Utility of Exome Sequencing for Kidney Disease. N Engl J Med. 2019;380(2):142–51.
4. Rasouly HM, Groopman EE, Heyman-Kantor R, Fasel DA, Mitrotti A, Westland R, et al. The Burden of Candidate Pathogenic Variants for Kidney and Genitourinary Disorders Emerging From Exome Sequencing. Ann Intern Med. 2019;170(1):11–21.
5. Landrum MJ, Lee JM, Riley GR, Jang W, Rubinstein WS, Church DM, et al. ClinVar: public archive of relationships among sequence variation and human phenotype. Nucleic Acids Res. 2014;42(Database issue):D980–5.
6. Sundaram L, Gao H, Padigepati SR, McRae JF, Li Y, Kosmicki JA, et al. Predicting the clinical impact of human mutation with deep neural networks. Nat Genet. 2018;50(8):1161–70.
7. Ioannidis NM, Rothstein JH, Pejaver V, Middha S, McDonnell SK, Baheti S, et al. REVEL: An Ensemble Method for Predicting the Pathogenicity of Rare Missense Variants. Am J Hum Genet. 2016;99(4):877–85.
8. Ren Z, Povysil G, Hostyk JA, Cui H, Bhardwaj N, Goldstein DB. ATAV: a comprehensive platform for population-scale genomic analyses. BMC Bioinformatics. 2021;22(1):149.
